# Five essential features for adoption of clinical risk prediction tools: Insights from the VOCAL-Penn score

**DOI:** 10.1097/HC9.0000000000000848

**Published:** 2025-12-01

**Authors:** Kinza Khan, Mackenzie Bolas, Nadim Mahmud

**Affiliations:** 1 Department of Medicine, Perelman School of Medicine, University of Pennsylvania, Philadelphia, Pennsylvania, USA; 2 Center for Clinical Epidemiology and Biostatistics, Department of Biostatistics, Epidemiology and Informatics, Perelman School of Medicine, University of Pennsylvania, Philadelphia, Pennsylvania, USA; 3 Division of Gastroenterology and Hepatology, Department of Medicine, Perelman School of Medicine, University of Pennsylvania, Philadelphia, Pennsylvania, USA; 4 Department of Medicine, Corporal Michael J. Crescenz Department of Veterans Affairs Medical Center, Philadelphia, Pennsylvania, USA; 5 Leonard David Institute of Health Economics, Perelman School of Medicine, University of Pennsylvania, Philadelphia, Pennsylvania, USA

**Keywords:** cirrhosis care, implementation framework, risk calculator, surgical risk assessment

## Abstract

**Background:**

Although medical risk prediction tools are widely developed, few achieve sustained clinical adoption. In cirrhosis patients, surgical risk calculators have achieved broad utilization. We sought to identify key design and implementation factors that influence provider uptake of such tools, using the VOCAL-Penn score (VPS) as a case example.

**Methods:**

We conducted a qualitative study of 22 diverse clinicians who care for patients with cirrhosis. Semi-structured interviews were guided by the Consolidated Framework for Implementation Research to explore factors influencing the adoption of risk prediction tools. Interviews were transcribed and analyzed using a combined grounded theory and deductive approach. Emergent themes were synthesized into a conceptual framework.

**Results:**

Five recurrent themes emerged as central: efficiency, accessibility, transparency, accuracy, and generalizability. Clinicians emphasized the need for tools that are intuitive, require minimal inputs, and integrate seamlessly into existing workflows. Ensuring input variables were clinically meaningful and readily available was cited as critical to encouraging use. Transparency in model development was essential to building trust. Participants stressed the importance of comparative performance data relative to existing clinical standards, as well as published external validations, to support the tool’s credibility. Finally, generalizability was key to equitable application across diverse patient populations.

**Conclusions:**

Using the VPS as a grounding example, our findings identify 5 domains—efficiency, accessibility, transparency, accuracy, and generalizability—that inform the development and dissemination of future tools. By aligning tool design with real-world clinical needs, this framework may support broader adoption and more equitable implementation of medical risk prediction tools.

## INTRODUCTION

Medical prediction models are increasingly employed to support clinical decision-making, particularly in complex or high-risk scenarios where accurate risk stratification is critical. Advances in data science, electronic health records, and machine learning have accelerated the development of these tools, resulting in the rapid proliferation of risk calculators spanning a range of medical specialties.^1^ However, relatively few prediction models are widely integrated into routine clinical practice. This implementation gap extends beyond limitations in statistical performance. While metrics such as calibration and discrimination are essential for all prediction models,^2^ successful adoption in practice often hinges on pragmatic factors—such as usability, accessibility, and credibility—that are seldom addressed during model development. As a result, even rigorously validated tools may remain underutilized in clinical settings.

The VOCAL-Penn score (VPS), developed in 2021, estimates postoperative mortality and decompensation risk among patients with cirrhosis undergoing surgical procedures.^3^,^4^,^5^,^6^ Since its introduction and external validation, the score has been widely adopted, with over 15,000 users annually, and has been incorporated into major national and international society guidelines. Despite this apparent real-world uptake, little is known about the contextual or design features that have supported its adoption, or how clinicians perceive and engage with the tool in clinical practice.

To better understand factors driving the real-world adoption of clinical prediction models, we examined the VPS as a case example. We aimed to explore how clinicians interact with such tools and to identify design and contextual factors that influence implementation. Grounded in implementation science, our goal was to generate practical, clinician-informed insights to guide the development of future models that are not only statistically sound but also usable, interpretable, and seamlessly integrated into clinical workflows.

## METHODS

### Study design and subject selection

This was a qualitative study with semistructured interviews of diverse clinicians involved in the risk assessment and stratification process for patients with cirrhosis facing a surgical decision. Purposeful sampling was used to identify a minimum target of 12–15 clinicians, given that this sample size is sufficient for thematic saturation based on established literature, targeting surgeons, gastroenterologists, hepatologists, and hospitalist physicians. For each clinician subject that was enrolled, data were collected regarding demographics (age, sex, race/ethnicity), practice setting, medical specialty, title (eg, fellow, instructor, assistant professor, etc.), and years in practice. Subjects were also asked about their prior familiarity with the VPS and whether they considered themselves to be regular users of the tool.

### Semistructured interview framework and conduct of subject interviews

The semistructured interview was designed following principles of the Consolidated Framework for Implementation Research (CFIR).^7^ The CFIR is a conceptual framework used in implementation science that serves as a guide for systematic evaluation of factors that may influence the implementation and/or effectiveness of a tool or intervention. There are 5 major CFIR domains that were applied to the design of interview questions in this study: (1) characteristics of the VPS calculator (eg, performance metrics, simplicity, etc.), (2) inner setting (eg, compatibility of the calculator with existing workflow, leadership advocacy), (3) outer setting (eg, patient needs, competing interventions, external incentives), (4) characteristics of individuals involved in using the tool (eg, practitioner beliefs regarding the efficacy of the calculator and ability to implement, patient perspectives regarding communication of projected risk), and (5) implementation process (eg, strategies to implement, identifying advocates). The semistructured interview script is provided in the Supplemental Material, http://links.lww.com/HC9/C165. Each clinician subject underwent an approximate 30-minute interview that was conducted over a video conferencing platform. At the outset of the interview, the clinician was shown a demonstration of the VPS web application calculator to ensure comprehension and grounding of the subsequent interview questions. All interviews were recorded and transcribed.

### Descriptive statistics and thematic analysis

Descriptive statistics for subject demographic and clinical practice data were summarized as medians and interquartile ranges (IQRs) for continuous variables and as frequencies and percentages for categorical data. For qualitative data, an integrated approach to data analysis was used to identify emergent themes from transcribed interviews.^8^ This entailed 2 approaches to reviewing and coding interview data. In the first, the grounded theory approach was utilized,^9^ where 5 transcripts were reviewed in careful detail to create coding constructs through induction.^10^ In the second, a deductive approach was used, where an a priori organizing framework using constructs from the CFIR and implementation outcomes of feasibility, acceptability, and utility were used to create codes.^11^ Two coders independently applied both inductive and deductive codes to transcripts and refined the codebook through iterative consensus. Coding disagreements were discussed in structured meetings, with unresolved discrepancies adjudicated by a senior author (Nadim Mahmud). To ensure reliability, a subset of additional transcripts was coded by both coders to confirm stability of the coding scheme. The finalized codebook was then applied to the full dataset in NVivo 14 (Denver, CO), after which data were thematically summarized and synthesized into a conceptual framework for future implementation efforts.

### Ethical considerations

This study received Institutional Review Board approval from the Hospital of the University of Pennsylvania.

## RESULTS

### Cohort characteristics

A total of 22 clinicians were identified and interviewed for this study. Median age was 38 years (IQR 33, 53), 14 (64%) were male, 10 (45%) were White, 16 (73%) practiced primarily in an academic center, and 15 (68%) were hepatologists (Table 1). There was diverse representation across titles and practice stages [ie, 3 (14%) trainees, 8 (37%) junior faculty, 8 (37%) associate or professor-level clinicians, and 3 (14%) non-academic physicians]. The median years in practice was 10 (IQR 7, 22).

**TABLE 1:** Subject characteristics

Variable and category	N=22
Age (y), median (IQR)	38 (33–53)
Sex	
Male	14 (64%)
Female	8 (36%)
Race/ethnicity	
White	10 (45%)
Asian	9 (41%)
Black	2 (9%)
Hispanic	1 (5%)
Practice setting	
Academic center	16 (73%)
Academic center/Veterans Affairs	3 (14%)
Private practice	1 (5%)
Health system	1 (5%)
Hybrid—academic/health system	1 (5%)
Medical specialty	
Hepatology	15 (68%)
Gastroenterology	4 (18%)
Surgery	2 (9%)
Internal medicine	1 (5%)
Title	
Resident	1 (5%)
Fellow	2 (9%)
Instructor	1 (5%)
Assistant professor	7 (32%)
Associate professor	3 (14%)
Professor	5 (23%)
Non-academic physician	3 (14%)
Years in practice, median (IQR)	10 (7–22)
Prior familiarity with the VOCAL-Penn score	18 (75%)
Regular user of the VOCAL-Penn score	9 (41%)

### Major identified themes

Thematic analysis revealed 5 core features perceived as essential for clinical adoption of risk prediction tools: efficiency, accessibility, transparency, accuracy, and generalizability. These domains emerged across multiple interviews and reflected both facilitators and barriers to VPS use, as well as broader expectations for clinical prediction tools.

### Efficiency

Clinicians consistently emphasized the importance of tools that are quick, low-burden, and intuitive to use in real time. Beyond speed alone, participants noted the value of clear, concise inputs that reduce cognitive load and eliminate ambiguity.

*“There [are] not a lot of numbers that you have to put into this model… simplicity of entry and it’s just really easy to do.”*

*“Just having something that’s very easy to use, easy to find, something I can just Google very easily and plug a few things into.”*

*“…in a room with a patient, If I can get the score done in less than a minute and I don’t have to do any sort of transformation of any variables based on … different units … that calculator becomes incredibly useful.”*

Participants preferred tools with minimal input requirements (ie, fewer variables) and interfaces that returned results in under a minute. Ease of use was further enhanced by features like automatic unit conversion (eg, SI to conventional units), which helped eliminate friction and supported real-time decision-making in diverse clinical settings.

### Accessibility

Seamless access to the prediction model within existing workflows was consistently noted to be essential. Clinicians favored tools that were embedded within the electronic medical record (EMR) system and/or within trusted clinical resources—such as UpToDate or MDCalc—or easily retrievable via web search without login barriers.

*“I use UpToDate like a lot of physicians. I want it all in one place—MELD, [the VPS], Child–Pugh, everything at my fingertips.”*

*“I need a concise place to access this… if you can integrate into the EHR that will be great.”*

Free availability and mobile-friendliness further enhanced usability. Many clinicians reported using prediction tools on both desktop and mobile devices, with some preferring phones for convenience or communication.

*“For this surgery, I used just my phone and I took a screenshot [of the VPS output] and sent it to the surgeon right away… very easy… it depends where I am.”*

*“It has to fit on the screen—it can’t be multiple pages—just to make it easier to use.”*

Cross-platform functionality, particularly the ability to toggle between devices in time-sensitive scenarios, was perceived as a key enabler of regular use.

### Transparency

Clinicians emphasized the importance of transparency in model design and application. This included clear documentation of derivation and validation, as well as well-defined, clinically intuitive inputs. Models relying on ambiguous or subjective variables—such as the ASA score—were seen as harder to trust or apply consistently.

*“I would like to see how the calculator was developed … what population studies have been done for it…”*

*“… anesthesiologists are more attuned to what the ASA is … I as a non-anesthesiologist have to look that up and say, ‘is this really a two or three?’ This brings some variability in how people may be using this and what the results are.”*

Participants valued face validity—models that reflected how clinicians naturally think about risk were viewed as more trustworthy and usable. In contrast, black box models or tools that lacked interpretability raised concerns, particularly in high-stakes settings where reasoning must be explained.

*“I have confidence [in the VPS] … I feel like I understand the model… It includes the variables that I think would be relevant.”*

Transparency also supported communication. Tools that clearly outlined their inputs, outputs, and use cases were seen as more conducive to team-based decision-making and shared understanding.

### Accuracy

Participants consistently emphasized the need for models to demonstrate credible, clinically meaningful accuracy. Confidence in a tool’s validity was closely tied to its comparative performance and calibration, particularly in relation to established benchmarks.

*“I trust the [VPS] predictions because of knowing that [the VPS] was … more accurate than the Mayo risk score.”*

Perceived accuracy was further reinforced by external validation. Clinicians expressed greater confidence when a model had been tested beyond its original development setting and demonstrated reproducible performance.

*“I saw there was an external validation in other health systems that made me more confident in using [the VPS].”*

*“[Colleagues] would be very much in favor of any objective validated tool that was accurate in predicting outcomes for their patients.”*

These perspectives highlight that real-world adoption hinges not just on numerical performance, but on clinician trust in validation and reproducibility.

### Generalizability

Trust in prediction models also hinged on their perceived applicability to diverse patient populations. Some participants raised concerns about generalizability, particularly in relation to sex representation in the underlying dataset.

*“The VA is a population that’s more representative of men, and so there’s underrepresentation of women in the data. So any validation that can be performed where there’s additional information on women… [will be] helpful.”*

Participants also noted that understanding the scale and diversity of the model’s derivation cohort enhanced their confidence in its applicability, particularly when it reflected real-world patient populations.

*“…for a lot of our staff, the buy-in was really understanding the magnitude of patients that [were] used to derive [the VPS].”*

These reflections suggest that technical accuracy alone is not sufficient; clinicians also assess whether a model was built and validated in populations that resemble their own, which they see as essential for real-world applicability.

## DISCUSSION

**FIGURE 1:**
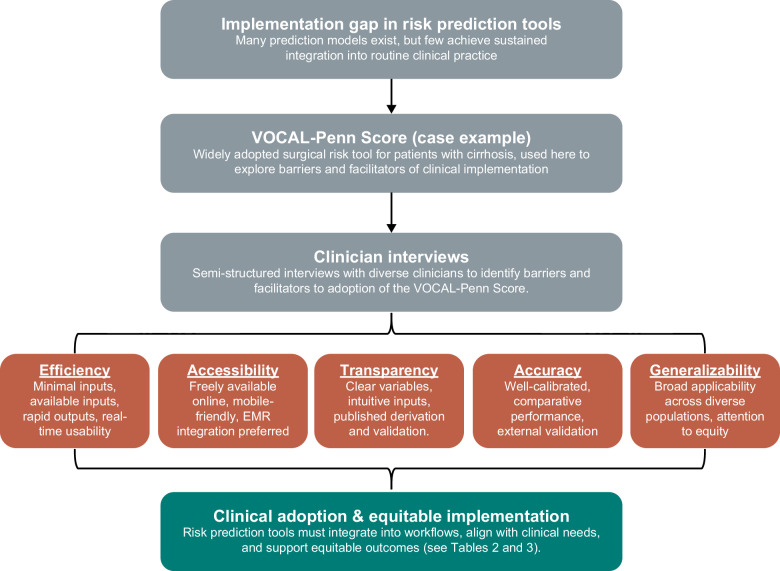
Conceptual model of VOCAL-Penn score implementation. Flow diagram illustrating the implementation gap in risk prediction tools, using the VOCAL-Penn score as a case example. Semistructured clinician interviews identified key barriers and facilitators to adoption, including efficiency, accessibility, transparency, accuracy, and generalizability, highlighting pathways toward clinical adoption and equitable implementation. Abbreviation: EMR, electronic medical record.

Despite the proliferation of clinical risk prediction tools, few are routinely used in everyday practice.^12^ In this qualitative study of clinicians using the VPS as a case example, we identified key factors that promote sustained use and successful clinical integration of prediction models (Figure 1). Participants consistently emphasized 5 domains—efficiency, accessibility, transparency, accuracy, and generalizability—as essential to clinical adoption. To illustrate how these domains map onto existing cirrhosis surgical risk tools, Table 2 compares the VPS with the Mayo Surgical Risk Calculator, Child–Turcotte–Pugh, and MELD-Na. This comparison highlights the strengths and limitations of each tool and demonstrates how specific characteristics influence clinician adoption. These themes, while grounded in cirrhosis, also reflect broader principles that may inform prediction model design and implementation across other clinical specialties.

**TABLE 2:** Comparison of risk prediction tools for surgical risk stratification in cirrhosis across 5 adoption domains

Domain	VOCAL-Penn score	Mayo Surgical Risk Calculator (MRS)	Child–Turcotte–Pugh score	MELD-Na
Efficiency	9 structured inputs; fast (~1 min); mix of routine labs and clinical variables, generally readily available	6 inputs; fast (~1 min); includes routine labs plus ASA and etiology, which require clinician entry	5 inputs; very fast (<30 s); combines routine labs with subjective clinical assessments (ascites, encephalopathy)	4 inputs; very fast (<30 s); all are objective routine labs
Accessibility	Freely available online; mobile-friendly; integrated into EMRs in some health systems	Available on the Mayo Clinic website; not widely embedded in EMRs or clinical platforms	Ubiquitous; embedded in EMRs, MDCalc, and widely available apps	Ubiquitous; built into EMRs, transplant systems, and online calculators
Transparency	Clear derivation and external validation; mostly objective inputs, though ASA introduces some inter-observer variability; accounts for surgery type	Published derivation and validation, but the etiology classification (alcoholic/cholestatic vs. viral/other) lacks face validity; it does not account for the surgery type	Includes subjective elements (ascites and encephalopathy grading), which may vary across clinicians	Objective lab-based inputs; INR may be confounded by anticoagulation, limiting interpretability in some patients
Accuracy	Strong discrimination and calibration; externally validated; outperforms Mayo, CTP, and MELD-Na for perioperative mortality and decompensation	Tends to overestimate perioperative mortality in the modern surgical era; not recalibrated for minimally invasive approaches	Good for global liver severity but not designed for perioperative outcomes; lacks surgical specificity	Robust for transplant-related mortality but not derived or calibrated for perioperative surgical risk
Generalizability	Derived from the national VA cohort but externally validated across multiple health systems; limited female representation	Derived from a single-center cirrhosis surgical cohort; limited external validation in contemporary diverse populations	Longstanding and widely validated for cirrhosis severity; perioperative use is based on secondary application rather than derivation for surgery	Widely validated in cirrhosis overall; perioperative use is based on secondary application rather than derivation for surgery

Abbreviations: EMR, electronic medical record; INR, international normalized ratio; VA, Veterans Affairs.

Our findings align with implementation science frameworks such as CFIR, underscoring the importance of compatibility between the tool, the clinical setting, and end-user needs.^7^ Participants highlighted the value of streamlined designs, pre-populated EHR data, real-time outputs, and clear variable definitions. These characteristics contributed to perceptions of trust and usability, reinforcing that predictive performance alone is not sufficient for uptake.^13^

To translate these insights into action, we propose a clinician-informed framework of pragmatic design considerations for future prediction tools (Table 3). This framework is organized around 4 key domains—usability and workflow integration, interpretability and decision support, validation and trust, and implementation and dissemination—each grounded in themes that emerged from clinician interviews. For example, participants emphasized the importance of tools that minimize data entry burden through EHR integration, deliver results in under a minute, and function across devices. They also highlighted the value of interpretable outputs linked to relevant clinical decisions, and the need for objective, routinely available inputs to support consistent use. Trust was bolstered when tools had undergone external validation and were calibrated to real-world populations.^12^ Finally, dissemination strategies such as embedding tools into EMRs or point-of-care platforms and providing training for end-users were seen as critical for uptake. Tools that neglect these principles—by relying on subjective inputs, operating in isolation from clinical workflows, or lacking clear utility—are unlikely to be adopted, regardless of statistical performance.^14^

**TABLE 3:** Pragmatic design and implementation considerations for clinical prediction models: Insights from VOCAL-Penn interviews

Category	Key considerations	Examples from VOCAL-Penn findings
Usability & Workflow Integration	Minimal data entry burden—Limit the number of required inputs to essential variables	Clinicians valued VOCAL-Penn’s relatively few required variables compared with other tools (eg, NSQIP)
	Pre-populated data from EHR when possible	Users wanted VOCAL-Penn integrated into EPIC with auto-populated lab values
	Quick and intuitive interface—Results should be obtainable in <1 minute	Speed was a major factor in model adoption
	Mobile-friendly and accessible across devices	Some users accessed VOCAL-Penn on phones for quick reference
Interpretability & Decision Support	Clear risk communication—Outputs should be actionable and meaningful for decision-making	The inclusion of decompensation risk was appreciated for postoperative planning
	Parsimonious model design—Include only essential predictors to balance simplicity and predictive accuracy	Although not directly discussed, minimizing inputs aligns with interview themes of usability
	Objective parameters—Favor measurable, reproducible variables to minimize inter-operator variability	Some providers expressed concerns about the subjectivity of ASA classification
	Use of routinely collected clinical data— Predictors should be easily accessible from routine clinical care	Concerns about NAFLD and subjective variables suggest a preference for readily available lab/imaging data
	Avoid predictors commonly confounded by clinical scenarios—Minimize use of variables affected by medications or temporary physiological changes	INR (impacted by warfarin) and creatinine (volume status) could lead to misleading risk estimates
	Comparison to existing gold-standard tools	Clinicians liked side-by-side comparisons with MELD, Child–Pugh, and Mayo risk scores
	Outcome relevance—Ensure predictions align with key clinical concerns	Participants wanted shorter-term (7-day) mortality estimates, not just 30-day outcomes
	Transparency in variable definitions—Avoid subjective or poorly defined inputs	Clinicians struggled with the ASA and NAFLD definitions in VOCAL-Penn
Validation & Trust	External validation across diverse populations	Some skepticism about VOCAL-Penn being VA-based without broader validation
	Comparison with real-world outcomes— Calibration should match observed results	Participants referenced needing “real-world” validation beyond development datasets
	Endorsement from professional societies and institutions	Users saw institutional buy-in as key to model uptake
Implementation & Dissemination	Integration into existing platforms—Embed in UpToDate, MDCalc, or EHRs	Many wanted VOCAL-Penn in MDCalc or EPIC dot phrases
	Training and education efforts—Provide guidance on model use, especially for non-specialists	Lack of awareness about VOCAL-Penn among non-hepatologists was a barrier
	Targeted dissemination to key users— Hepatologists, anesthesiologists, and surgeons	Clinicians emphasized spreading awareness via grand rounds, CME talks, and social media

Abbreviations: ASA, American Society of Anesthesiologists; CME, Continuing Medical Education; EPIC, Electronic Health Record system developed by Epic Systems Corporation; NSQIP, National Surgical Quality Improvement Program; VA, Veterans Affairs.

Our study findings align with established literature examining factors that influence the adoption of clinical decision support tools and risk prediction models. Across diverse contexts, common drivers of uptake include trust in the model (through transparency and accuracy) and seamless integration into existing workflows, while barriers often center on time burden, integration challenges, and perceived lack of relevance.^15^,^16^ Our results corroborate these themes but also extend them by elevating accessibility and generalizability to the same conceptual tier as transparency and accuracy—whereas prior studies typically treated these as secondary considerations rather than core elements of real-world adoption.^17^ In addition, this study contributes an explicit, interview-derived framework that translates clinician perspectives into concrete, implementable specifications, moving beyond the more general descriptions provided in earlier qualitative work.^18^,^19^

Our study results carry practical implications for model development and dissemination. Model developers should pair statistical refinement with iterative testing among diverse clinician users to identify design pitfalls early. Institutions should prioritize embedding tools into EHRs and trusted clinical resources to reduce workflow barriers. Finally, funders and journals might require explicit implementation strategies, ensuring that dissemination plans attend not only to predictive performance but also to practical deployment in the settings where clinicians and patients will actually use these tools.

There are important limitations that we acknowledge in our study. First, although we achieved thematic saturation, the sample was relatively enriched with hepatologists and academic physicians, which may limit generalizability to non-specialist or community-based clinicians. Second, participant familiarity with the VPS varied, and while all received a standardized demonstration, their differing baseline experience may have influenced responses. Third, because themes were derived through analysis rather than elicited for prioritization, we cannot determine the relative salience of different features (eg, accuracy vs. workflow integration) in influencing adoption. Future work should assess which factors most strongly drive use. Fourth, because the study focused on a single risk prediction model within a specific implementation context, findings may not fully translate to other tools or settings. Nonetheless, the consistency of themes across specialties suggests broader relevance. Finally, as with all qualitative work, the analysis is shaped by interpretive processes despite efforts to ensure rigor.

Effective prediction models must do more than calculate—they must integrate seamlessly into practice and inspire clinician trust.^20^ By grounding our recommendations in real-world perspectives, this study highlights pragmatic levers that can improve adoption, equity, and ultimately patient care.

## Supplementary Material

## Data Availability

The data, analytic methods, and study materials used in this study may be disclosed upon reasonable request. Dr Mahmud is supported by the National Institute of Diabetes and Digestive and Kidney Diseases (K08-DK-124577, R03-DK134794) and investigator-initiated funding from Grifols unrelated to this work. Mackenzie Bolas is employed by the University of Pennsylvania. Nadim Mahmud received grants from GRIFOLS.
