# Supplementary material for: Five essential features for adoption of clinical risk prediction tools: Insights from the VOCAL-Penn score
Source: Hepatol Commun. 2025 Dec 1;9(12):e0848. doi: 10.1097/HC9.0000000000000848 (PMC12668580; doi:10.1097/HC9.0000000000000848)
Supplement: Supplementary file 1 [file hc9-9-e0848-s001.docx]

**Supplement**

**Semi-Structured Interview Script**

Thank you for agreeing to talk to me today about the VOCAL-Penn cirrhosis surgical risk calculator. As you know, the VOCAL-Penn calculator is designed to aid clinicians in making decisions about surgery for patients with cirrhosis. Since you are likely to see patients with cirrhosis in your practice, we are interested in what you think about the calculator and we would like to understand how the VOCAL-Penn calculator might best meet the needs of clinicians like you. With your permission, I would like to audio record our conversation. After we are done, I will have the conversation transcribed and, in the process I will remove your name and identifying information that you may provide about yourself or others. May I have your permission to record our conversation?

Thank you.

**Script for VOCAL-Penn Website Demonstration**

I would like to briefly show you the website and demonstrate its features.

<*navigate to* [*www.vocalpennscore.com*](http://www.vocalpennscore.com)>

The intended use of the VOCAL-Penn Calculator is summarized in this box <*indicate to box on the lower right*>. For a patient with cirrhosis who is being considered for surgery, this tool can be used to predict the likelihood of death at 30, 90, and 180 days after surgery. This may help with patient selection and prognostication for surgeries. To use the calculator, the user inputs readily available clinical data for the patient in this area <*indicate to the box on the left*>.

For example, let’s say that we have a patient with the following information:

<*input the following data while talking*>

A patient with age 53 years, albumin 2.5, bilirubin 2.3, platelet count 130, a body mass index over 30, without non-alcoholic fatty liver disease, and with an American Society of Anesthesiology score of 4. The surgery of interest is non-emergent, and is an open abdominal surgery. When you click on ‘Surgery Type’ you will see that there are several categories of surgery that can be selected.

If you are unsure as to what surgeries are included in different categories, you can click on ‘**Surgery Types**’ <*click on this tab in the lower right box and scroll through*> and scroll to see common examples.

Additionally, if you are unsure of the meaning of an input, you can click on the blue text <*demonstrate* *with ASA*>. For example, for ASA input, the meanings of the numeric scores are summarized here.

Once you are ready, click on ‘**Calculate**’ and the tool will display the risk of post-operative mortality here <*indicate to box on the upper right*>.

If you would like to copy the results into a note, you can click on the ‘**Copy**’ button and then paste the results wherever you would like.

**Baseline Information and Prior Experience**

I would like to start by gathering a little information about you.

- If you are caring for a patient with cirrhosis who is being considered for surgery, what is your typical approach to risk stratifying the patient? Do you use any specific tools or calculators in this process? Do you typically encounter this scenario more often in the inpatient or outpatient setting?
- Have you ever used the VOCAL-Penn calculator before?
  - If yes, how did you hear about the calculator? What was the scenario in which you used the tool? What was your initial experience with the VOCAL-Penn calculator?
- If you use a risk stratification tool, how do you access it (i.e. computer, phone, tablet)?

**Intervention Characteristics**

(I understand you may be familiar with the tool, however) I would like to briefly demonstrate the VOCAL-Penn calculator and show you its features.

- What do you think about this VOCAL-Penn calculator as a surgical risk prediction tool?
- What would you need to know in order to have confidence in the predictions generated by the tool?
- How does this tool compare to other tools you might use to assess surgical risk for people with cirrhosis?
- What features of the calculator make this attractive to you?
- What features of the calculator make it unattractive to you?

**Inner Setting**

- What do you think about the way that surgical decisions for patients with cirrhosis are made in your practice?
  - What influences the ways that surgical decisions are made?
- Can you recall a scenario where a patient was appropriately risk stratified for surgery at your practice? Can you describe that scenario?
- Can you recall a scenario where a patient was inappropriately risk stratified? Please describe that scenario.
- How does use of the VOCAL-Penn score align (or not align) with the goals of your practice or institution?

**Process**

- How might a tool like the VOCAL-Penn calculator be integrated into a practice or institution’s workflow?
- Who would be the biggest advocates for a tool like the VOCAL-Penn calculator?
- How would you advocate for the VOCAL-Penn calculator, or go about engaging others to use this tool?
- How could the VOCAL-Penn calculator be improved to simplify incorporation into practice?

**Outer Setting**

- What do you think professional organizations (for example, medical societies such as the American College of Gastroenterology or the American Association for the Study of Liver Diseases) would say about using risk prediction tools like the VOCAL-Penn calculator?
- What do you think patients with cirrhosis and their families would think about a tool like the VOCAL-Penn calculator?
- What do you think others in your field at other institutions think about a tool like the VOCAL-Penn calculator?
  - What have you seen them do to aid in making decisions about surgery for patients with cirrhosis?

Thank you for sharing your ideas with me. Is there anything else about the tool or about the risk stratification process for surgery for people with cirrhosis that you think I should know?

Finally, I would like to briefly obtain some basic information about yourself and your clinical practice.

- May I ask your age and self-identified sex and race?
- What is your medical specialty? What is your current title or position? How many years have you been in practice, either as a trainee or as an independent provider? What is the name of the institution where you currently practice?
- What type of setting do you practice in, for example private practice, community hospital, or academic center? Approximately what percentage of your current position is spent on clinical work?
